# Analysis of evolution and genetic diversity of sweetpotato and its related different polyploidy wild species *I. trifida* using RAD-seq

**DOI:** 10.1186/s12870-018-1399-x

**Published:** 2018-09-05

**Authors:** J. Y. Feng, M. Li, S. Zhao, C. Zhang, S. T. Yang, S. Qiao, W. F. Tan, H. J. Qu, D. Y. Wang, Z. G. Pu

**Affiliations:** 1 Biotechnology and Nuclear Technology Research Institute, Sichuan Academy of Agricultural Sciences, Chengdu, 610061 China; 2 Center of Analysis and Testing, Sichuan Academy of Agricultural Sciences, Chengdu, 610061 China; 3 Crop Research Institute, Sichuan Academy of Agricultural Sciences, Chengdu, 610066 China

**Keywords:** Sweetpotato, RAD-seq, SNP, SSR, Evolution analysis

## Abstract

**Background:**

Sweetpotato (*Ipomoea batatas* (L.) Lam.) is one of the most important crops from the family of *Convolvulaceae*. It is widely reported that cultivated sweetpotato was originated from *Ipomoea trifida*. However, diploid, tetraploid and hexaploid *I. trifida* were found in nature. The relationship, between them, and among them and sweetpotato, is remaining unclear.

**Results:**

In the present study, we detected the genome diversity and relationship of sweetpotato and different polyploidy types *I. trifida* using Restriction-site Associated DNA Sequencing (RAD-seq). A total of 38,605 RAD-tags containing 832,204 SNPs had been identified. These tags were annotated using five public databases, about 11,519 tags were aligned to functional genes in various pathways. Based on SNP genotype, phylogenetic relation analysis results confirmed that cultivated sweetpotato has a closer relationship with *I. trifida* 6× than with *I. trifida* 4X and *I. trifida* 2×. Besides, 5042 SSRs were detected in *I. trifida* 6×, and 3202 pairs of high-quality SSR primers were developed. A total of 68 primers were randomly selected and synthesized, of which 61 were successfully amplified.

**Conclusion:**

These results provided new evidence that cultivated sweetpotato originated from *I. trifida* 6×, and that *I. trifida* 6× evolved from *I. trifida* 4X and *I. trifida* 2×. Therefore, using *I. trifida* 6× as the model plant of sweetpotato research should be more practical than using *I. trifida* 2× in the future. Meanwhile, sequence information and markers from the present study will be helpful for sweetpotato and *I. trifida* studies in the future.

**Electronic supplementary material:**

The online version of this article (10.1186/s12870-018-1399-x) contains supplementary material, which is available to authorized users.

## Key message

We report a genome-wide SNP&SSR discovery and SSR marker development using RAD-seq, and phylogenetic analysis of sweetpotato and different polyploidy of *Ipomoea trifida*.

## Background

*Ipomoea*, including about 600–700 species, has maximum genus in the family of *Convolvulaceae* [1]. About 13 wild species of this genus belong to *Ipomoea* section *Batatas*. Sweetpotato (*Ipomoea batatas* (L.) Lam.) is the only species that is widely cultivated as a major staple crop in over 100 countries [2]. It plays an important role in food security in numerous African countries [1, 3]. However, the study of sweetpotato in genetics and genomics lags far behind that of other major crops for its complex genome structure and cross incompatibility [4].

So far, the ancestor of sweetpotato and its domestication remained unclear. Only few studies about the relationships between sweetpotato and its wild species have been reported [5, 6], but some debate about sweetpotato origins still exists. It is widely accepted that *I. trifida* is the closest wild species of sweetpotato [7, 8]. However, the genetic relationships between sweetpotato and its wild species have not been fu1ly elucidated.

Wild relative species of sweetpotato possess lots of desirable traits, such as drought and salinity tolerance, disease resistance, and high content of starch, etc.. They should be a precious gene reservoir for sweetpotato breeding and cultivar improvement, and may provide a new approach for sweetpotato genetic study. Therefore, it is crucial to elucidate the genetic relationships between sweetpotato and its allied species in the future genetic studies.

Several hypotheses have been applied to reveal the origin of sweetpotato. Recent studies were focused on two perspectives about the origin of cultivated sweetpotato [5, 9, 10]. The first hypotheses proposed the ancestor of sweetpotato was from a cross between *I. trifida* and *I. triloba*. Another hypothesis proposed that a hybridization between diploid *I. leucantha* and its polyploidization tetraploid *I. littoralis Blume* generated triploid *Ipomoea trifida* (H.B.K.) Don., which spontaneous polyploidy originated wild ancestor of hexaploid *I. batatas*. Although two hypotheses supported different origin models of sweetpotato, they all acknowledged that *I. trifida* is one of the ancestors of sweetpotato.

*I. trifida* (H. B. K.) G. Don. has a complex forms of polyploidy, including diploid (2 *N* = 2× = 30), triploid (2 *N* = 3× = 45), tetraploid (2 *N* = 4× = 60), and hexaploid (2 *N* = 6× = 90), and it is cross-compatible with cultivated sweetpotato [5]. Furthermore, several studies in molecular genetics and cytogenetic indicated that *I. trifida* is the closest wild relative of cultivated sweetpotato [7], and it was considered to be the most likely candidate progenitor of sweetpotato.

With the application of next-generation sequencing (NGS) technology, genomics has developed rapidly in recent years. More and more whole-genome sequences of plants and animal species have been released [11]. However, many agricultural crops and animals were still without reference genomes because of the complexity of genome. The situation will be hard to change in the near future. For these species, reduced-representation libraries sequencing (RRLS) is deemed to be the most effective and economic choice in genomics research [12–14]. Restriction-site Associated DNA sequencing (RAD-seq) is one of the widest used RRLS techniques, which combines the advantages of low cost and high throughput [15], and it is particularly useful for genome studying in species lacking reference genomes. Now, RAD sequencing was widely used in genetic diversity, ecological and evolutionary genetics, genetic mapping and molecular markers development studies [16, 17].

In this study, we used RAD-seq to (1) evaluate the use value of this approach, (2) detect plenty of SNPs in sweetpotato and its related wild species *I. trifida*, (3) evaluate the phylogenetic relationships among sweetpotato accessions and different polyploidy forms of *I. trifida*, (4) detect SSR loci in *I. trifida* 6× and develop SSR markers. These results will be helpful to understand the phylogenetic relationship and genetic diversity of *I. batatas* species and its putative progenitor *I. trifida*.

## Results

### RAD-seq and SNP discovery

By sequencing the genomes of 27 samples, including sweetpotato cultivars, *I. trifida* (2×, 4X, 6×) and synthetic accessions, a total of 37.29 Gb high-quality sequence data, containing 100,507,572 pair-end reads and 7,843,552 single-end reads, was obtained. The number of raw reads from each accession ranged from 6,453,618 to 23,422,064 with an average of 9,320,100. Although genomes of sweetpotato and *I. trifida* 6× were larger in size than *I. trifida* 2× and *I. trifida* 4X, the total number of raw reads had no significant difference. The number of total raw reads was 45,236,943. The raw reads number for sweetpotato ranged from 2,545,682 to 9,994,361 with an average of 3,831,194. For different polyploidy type *I. trifida*, *I. trifida* 2× had an average raw reads of 3,965,810, and average raw reads of 3,511,641 were for *I. trifida* 4X, and 2,975,540 raw reads were from *I. trifida* 6×. After filtering the raw reads, 8,585,422 high-quality reads-tags were generated. For each sample, number of reads-tags ranged from 209,850 to 608,047, with an average of 317,978 (Tables 1 and 2).

**Table 1:** The statistic results of RAD sequencing raw reads in 27 accessions

	Read number	Read1 length	Read2 length	Total bases	Q20	Q30
Min.	6,453,618	144.00	151.00	955,135,464	92.63	84.21
Max.	23,422,064	147.00	151.00	3,478,176,504	95.59	89.53
Total	251,642,702	–	–	37,286,365,861	–	–
Average	9,320,100	145.37	151.00	1,380,976,513.37	93.77	86.21

**Table 2:** The statistic of RAD-tag number, total reads and sequencing depth in 27 accessions

	RAD-tags number	Total reads	Seq. depth	Pair reads number	Single reads number	Total bases (bp)
Min.	209,850	2,616,331	8.60	2,454,248	162,083	2,486,118,960
Max.	608,047	10,596,916	21.98	10,144,386	452,530	616,953,960
Total	8,585,422	108,351,124	328.10	100,507,572	7,843,552	25,063,043,520
Average	317,979	4,013,005	12.15	3,722,502	290,502	928,260,871

After grouping RAD-seq reads into RAD-tags, the sequencing depth was identified as varying significantly across loci, which has been observed in many other RAD-seq based studies. The majority of the loci were in coverage between 8.6 and 21.98. The mean coverage of polymorphic loci was 12.15 (Additional file 1: Figure S1), which was at a medium level (Table 2).

All candidate alleles identified among cultivated sweetpotato and different polyploidy types of *I. trifida* were clustered using SNP genotype. Finally, 38,605 RAD-tags were comparable among 27 samples, and 832,204 SNPs had been identified. The A/G type, T/C type and A/C type SNPs were accounted for 32.87%, 29.17% and 13.34%, respectively.

### Genetic relationship between sweetpotato and *I. trifida*

**Fig. 1:**
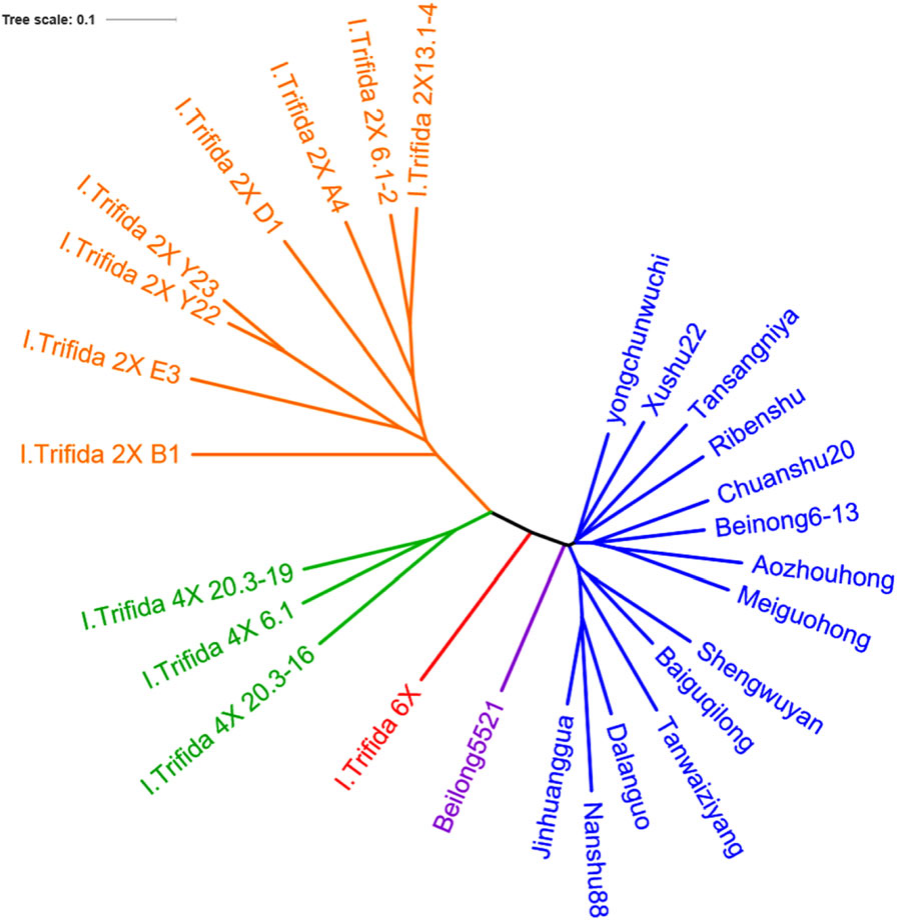
Phylogenetic tree of 27 accessions based on identified SNP

**Fig. 2:**
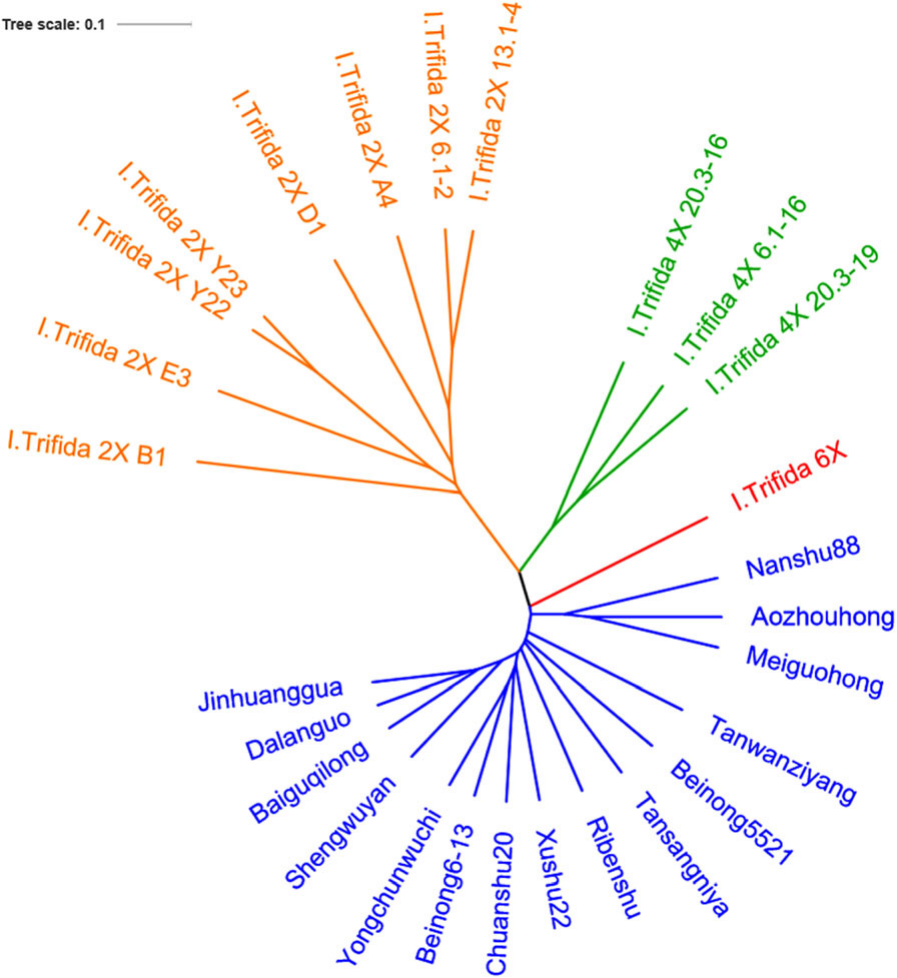
Neighbor-joining trees showing the genetic relatedness of the 27 accessions

The application of Phylip in combination with PLINK and fastSTRUCTURE revealed evolutionary history and population structure across 27 accessions of *I. batatas* and *I. trifida*. Phylogenetic analyses of combining data matrix from all SNPs showed that the trees constructed with parsimony genetic distances and neighbor-joining had almost the same topologies (Figs. 1 and 2). Twenty-seven accessions tested in this study were grouped into three major groups at the distance of 0.02884. The first group, including 13 sweetpotato accessions and one synthetic line Beinong 6–13, could be clearly separated into three clusters. Five Chinese sweetpotato landraces Baiguqilong, Shengwuyan, Dalanguo, Jinhuanggua, Tanwanziyang and a modern variety Nanshu88 were clustered together. Another cluster contained one Japanese cultivar, one Tanzanian cultivar and two Chinese cultivars. The last cluster included two accessions Meiguohong from U.S. and Aozhouhong from Australia, and one Chinese modern cultivar Chuanshu20 and synthetic line Beilong 6–13 (Fig. 1). The second group contained eight *I. trifida* 2X accessions. These accessions had the distinct characteristics of *I. trifida* 2X, such as thin stem, small leaf small seed. Moreover, the accessions in this group all have 30 chromosomes based on cytological observation. The third group, which located between the first group and the second group, consisted of three *I. trifida* 4X accessions, one *I. trifida* 6× accession and one synthetic line Beilong 5521 (Fig. 1). The phylogenetic tree revealed that *I. trifida* 6× are more closely related to *I. trifida* 4X than to *I. trifida* 2X.

Phylogeny was constructed based on genetic similarity calculating using SNP datasets. The SNPs-based UPGMA genetic similarities among each accession ranged from 0.1779 to 0.06699. A dendrogram was constructed based on the simple matching coefficient. At the distance of 0.02295, two groups were clustered in the dendrogram, and two distinct sub-groups existed in one group, which almost the same as phylogenetic analyses results (Fig. 2).

Group 1 contained all eight *I. trifida* 2X, but genetic similarities were different among them. *I. trifida* 2X B1 showed the biggest difference with other genotypes. *I. trifida* 2X Y23 and *I. trifida* 2X Y22, had the closest relationship with a genetic similarity 0.05087, followed by *I. trifida* 2X 13.1–4 and *I. trifida* 2X 6.1–2 with a genetic similarity 0.08058. There were abundant in genetic diversity among each *I. trifida* 2X in this group. The second group included three *I. trifida* 4X accessions, one *I. trifida* 6X and all sweetpotato accessions. Two distinct sub-groups could be identified in this group. One sub-group only contained three *I. trifida* 4X accessions. Interestingly, the other sub-group consisted of one *I. trifida* 6X and all sweetpotato accessions. It showed *I. trifida* 6X had a closer relationship with *I. batatas* than that with *I. trifida* 4X and *I. trifida* 2X. Except *I. trifida* 6X, there was a little genetic difference in this sub-group. In contrast, Nanshu88, Meiguohong and Aozhouhong showed the difference with extra sweetpotato accessions. Two of them came from America and Australia, respectively, and Nanshu88 was a widely grown Chinese sweetpotato verity. The other sweetpotato accessions had high genetic similarity with each other in the dendrogram. In addition, the dendrogram showed that synthetic species Beinong5521 and Beinong6–13 were closely related to sweetpotato as compared to other wild species (Fig. 2).

**Fig. 3:**
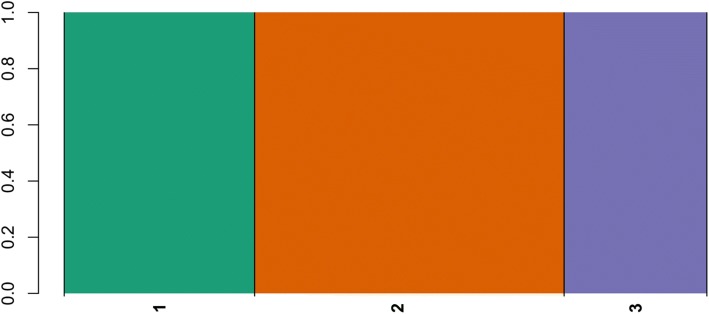
Population structure analysis based on identified SNP

To further reveal the genetic structure, fastSTRUCTURE software was utilized with different K values from 1 to 10 based on all effective SNP markers. Using the model-based clustering analysis, the optimal number of clusters to describe the data was K = 3, which distinguished *I. trifida* 2X, cultivated *I. batatas* accessions and *I. trifida* 6X, cultivated *I. batatas* accessions and *I. trifida* 4X. This grouping result also basically confirmed the phylogenetic analyses results and genetic similarity. *I. trifida* 2X accessions were represented by cluster K1. Three accessions of *I. trifida* 4X were clustered in K3, in addition, K3 contained 3 sweetpotato accessions, namely Nanshu88, Danlanguo and Jinhuanggua. *I. batatas* accessions were grouped to clusters K2 and K3. Except three *I. batatas* accessions were attributed to *I. trifida* 4X cluster (K3), most of the *I. batatas* accessions were clustered into K2, which included landraces and modern cultivars. Interestingly, *I. trifida* 6X and two synthetic species also were divided into K2 group. Consequently, clustering results indicated the genetic relationship between *I. batatas* and *I. trifida* 2X is quite far from the phylogenetic relationship of *I. trifida* 4X and *I. trifida* 6X. By contrast, genetic distances revealed that *I. trifida* 6X has a closer genetic relationship with sweetpotato than with *I. trifida* 4X and *I. trifida* 2X (Fig. 3).

### SSR loci detection in *I. trifida* 6X

**Fig. 4:**
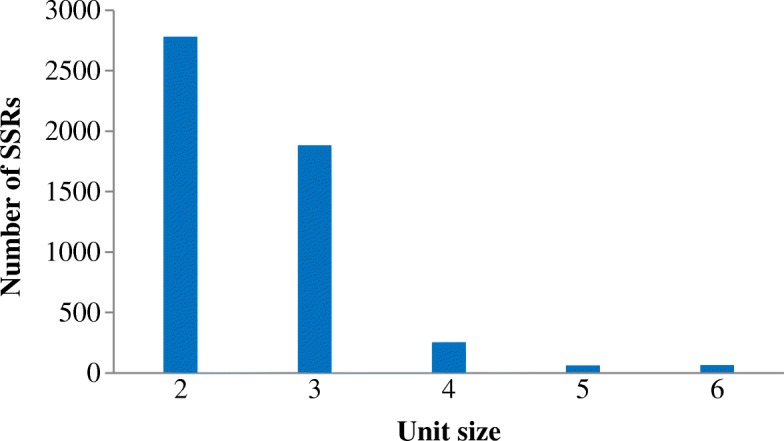
The distribution SSRs loci number with different unit size

To further detecting SSR loci, the MISA script (http://pgrc.ipk-gatersleben.de/misa/) with the default settings was used to analyze in *I. trifida* 6X. The criteria included a 50 bp minimum match, 95% minimum identity in the overlap region and 20 bp maximum unmatched overhangs. The results indicated a total of 5042 SSRs were identified from 55,167,516 bp of 180,286 reads in *I. trifida* 6X, with an average of one SSR per 3.6 kb. 2951 SSR contained sequences (Table 3). In total, the compilation of all SSRs revealed that the proportion of SSR unit sizes was not evenly distributed. Among all SSRs, 2779 (55.12%) SSRs were belonging to 2 unit size types, followed by tri-nucleotide repeat motifs, accounting for 1882 (37.33%). A total of 381 (7.56%) SSRs had unit size between 3 to 6 (Additional file 2: Table S1) (Fig. 4). In total, 1812 sequences containing more than 1 SSR, and 694 SSRs presented in compound formation that have more than one repeat type (Table 3).

**Table 3:** The detecting results of SSR loci in *I. trifida* 6X

Item	Number
Total number of sequences examined	180,286
Total size of examined sequences (bp)	55,167,516
Total number of identified SSRs	5042
Number of SSR containing sequences	2951
Number of sequences containing more than 1 SSR	1812
Number of SSRs present in compound formation	694

Among 5042 SSRs, 4172 SSRs were highly dispersed with sizes ranging from 10 bp to 20 bp, which accounted for 82.74% of total SSRs, followed by 21–30 bp (715 SSRs, 14.18%) (Additional file 2: Table S1). A maximum of 69 bp Tri-nucleotide repeats (TAA) was observed. In addition, a total of 224 SSR motifs were identified, of which, di-, tri-, tetra-, penta- and hexa- nucleotide repeat had 4, 10, 31, 67 and 112 types, respectively. The AT/TA di-nucleotide repeat was the most abundant motif detected in RAD sequences (779, 15.45%), followed by the motif GA/AG (721, 14.30%), TC/CT (696, 13.80%), TG/GT (298, 5.91%), AC/CA (279, 5.53%), AAT/TAA (93, 4.01%) and TTC/CTT (166, 3.29%). The frequency of remaining 161 types of motifs accounted for 3.19% (Fig. 4).

### Development and evaluation of SSR markers

Based on the sequences of 5042 SSRs, 3202 pairs of high-quality SSR primers were successfully designed after stringent filtering using Primer Premier 5.0 (PREMIER Biosoft International, Palo Alto CA). To further evaluate the polymorphism of SSR markers, 68 SSR markers were randomly selected and synthesized. After being tested by 3 *I. trifida* accessions, 61 primer pairs (89.71%) were successfully amplified. Different annealing temperatures were tried, 7 (10.29%) SSR primers still were unable to generate PCR products. Most of these 61 working primer pairs amplified bands almost at the expected sizes, except for 11 SSR primers amplified with larger bands than that expected.

**Fig. 5:**
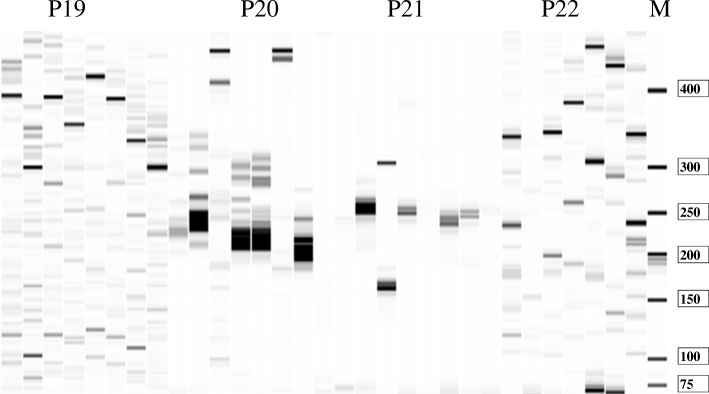
The result of capillary electrophoresis. P12–22 was novel SSR primers pairs. M was a marker. The accessions in each group were different *I. trifida* genotypes from Venezuela, Mexico and Colombia

The polymorphism of the 61 SSR primer pairs was further evaluated in eight diverse accessions of *I. trifida* (Fig. 5). The results showed that all primers amplified more than two bands and all primers had polymorphic bands. PCR products ranged in size from 100 bp to 480 bp. The number of polymorphic loci varied from 2 to 11 per optimized primer, with an average of 6 polymorphic loci.

### Annotation of sequences with SNPs

Total of 38,605 sequences with polymorphic SNP loci were blasted using Blast2GO software, of which 11,519 (29.84%) matched significant hits (E = 10^− 5^) in Nr database. In total, 11,541 (29.90%) of them aligned with sequences in UnProtKB database, including 2516 reads only with blast hit, and 2551 hits were mapped without GO Annotation. Only 6474 (16.77%) hits were successfully mapped and annotated. 27,064 (70.10%) singletons had no significant hits. The frequency of sequences with significant hits was lower in the present study than that in transcription studies. It could be because most reads without hits were derived from non-coding regions of the genome.

The number of hits was over 50 in 48 species, most of which belonged to biannual or perennial plants. 1726 reads were hit in *Ipomoea nil*. 866 and 722 were hit in *Nicotiana sylvestris* and *Nicotiana tomentosiformis*, respectively. Interestingly, only 200 hits were from *Ipomoea batatas*, and 127 hits were from *Ipomoea trifida*. In addition, only few hits were from other *Ipomoea* species, including *Ipomoea purpurea*, *Ipomoea pes-caprae*, etc. (Additional file 1: Figure S2).

**Fig. 6:**
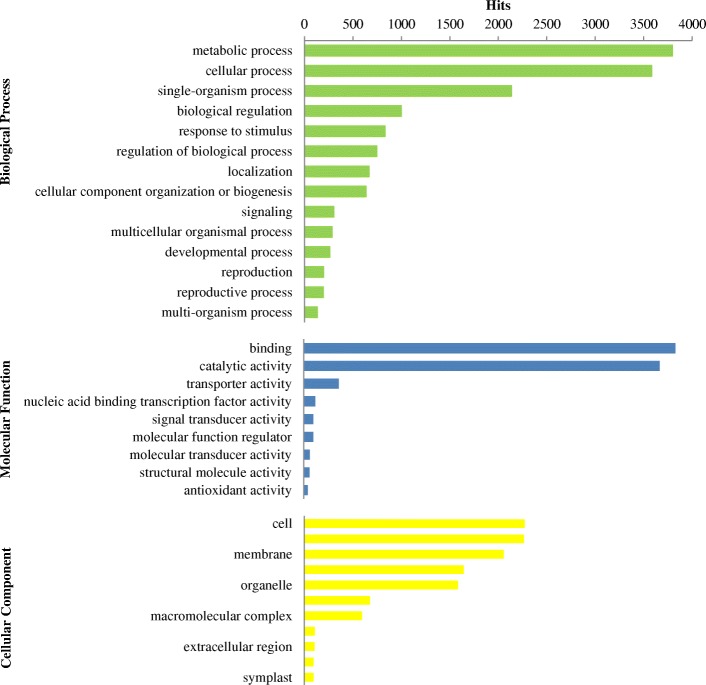
The distribution of Gene Ontology (GO) classification with different annotations

A total of 6474 (16.77%) out of the 38,605 blastx-annotated sequences could be associated with GO terms. Totally 35,384 unigenes were annotated. Based on annotation analysis, the blast hits genes were divided into molecular function (MF), cellular component (CC), and biological process (BP) categories at level 2. 15,163 (42.85%) annotations related to biological process, 8328 (23.54%) reads connected to molecular function, and 11,893 (33.61%) reads worked in cellular component. In biological process, ‘metabolic process’ (3800), ‘cellular process’ (3588) and ‘single-organism process’ (2142) were prominently represented. For the molecular function category, ‘binding’ (3803), ‘catalytic activity’ (3641) and ‘transporter activity’ (358) were the most highly represented categories. Under cellular components category, the largest proportion of genes were divided into ‘cell’ (2334), ‘cell part’ (2324) and ‘membrane’ (2110) (Fig. 6). Additionally, only 7 sequences were assigned to ‘nutrient reservoir activity’ terms.

From analysis GO evidence codes, we detected 8836 (72.47%) putative genes inferred from Electronic Annotation (EA). These annotations have not been manually checked. 1749 (14.34%) putative genes were derived from Biological Aspect of Ancestor (BA). The remaining 1148 (9.42%) putative genes were inferred from expression pattern (EP), direct assay (DA), mutant phenotype (MP), Sequence Model (SM), and Sequence or structural similarity (SS) (Additional file 1: Figure S3).

In parallel, a total of 8587 RAD-tags, were aligned to OG databases using Blast2GO. Interestingly, 2025 (23.58%) of them were with unknown function. This may be related to the reads in the present study were from genome, which had non-coding region, or because there are few studies on sweetpotato and *I. trifida*. 6562 sequences were annotated in OG categories. ‘Signal transduction mechanisms’ and ‘Posttranslational modification, protein turnover, chaperones’ had the largest amount sequences of 992 (11.55%) and 703 (8.19%), respectively, followed by ‘Energy production and conversion’, ‘Replication, recombination and repair’ and ‘Carbohydrate transport and metabolism’, with 671 (7.81%), 558 (6.50%) and 495 (5.76) sequence, respectively (Additional file 1: Figure S4). About 164 (1.91%) sequences were annotated with ‘Defense mechanisms’, which should have particular potentials for resistance gene study in sweetpotato and *I. trifida*.

Mapping of genes for putative proteins onto the KEGG database was performed along with e value <=1e^− 5^ and rank <=5. In the pathways categorized ‘Metabolism’ in the KEGG database, a total of 1727 unigenes were mapped onto 349 metabolic pathways (Additional file 1: Figure S5). The pathways included ‘Starch and sucrose metabolism’, ‘Carotenoid biosynthesis’, ‘Brassinosteroid biosynthesis’, ‘Photosynthesis-antenna proteins’, ‘Indole alkaloid biosynthesis’, ‘Photosynthesis’, ‘Zeatin biosynthesis’, ‘Biosynthesis of amino acids’, ‘Plant hormone signal transduction’, which should provide valuable information for future studies.

## Discussion

### RAD-seq in sweetpotato and *I. trifida*

The next generation (NGS) sequencing can be used to infer evolutionary relationships between species, as well as identify a large number of SNPs scattered among the genome. In this study, we sequenced sweetpotato and different polypoid types of *I. trifida*, and found about 832,204 SNP loci and 5042 SSRs. These markers could be broadly used for mapping agronomic genes and constructing genetic maps in sweetpotato and *I. trifida*.

The genetic complexity species without reference genome are still difficult to carry out genetic study using advantage of NGS, the situation is difficult to change in the near future. Some researchers have attempted to assemble preliminary reference genomes at first, followed by re-sequencing to identify a set of SNPs. However, this approach is too costly to carry out in the majority of species. On the compromise of SNP quality and reduce the cost, several methods have been tried. Finally, reduced-representation libraries sequencing (RRLS) was set up, which offered a greatly simplified library production procedure more amenable to use on large numbers of individuals/lines [18]. Now, several RRLS methods have developed, including Restriction site Associated DNA (RAD) sequencing [16, 19], and Genotyping by Sequencing (GBS) [20, 21], etc.. Using RRLS to study genetic complexity species without reference was much more efficient and cheap.

Numerous reports indicated that RAD-seq data can be utilized to accurately determine SNP genotype without a reference genome [22, 23]. However, accurate results were built on relatively elevating sequencing depths. Nonetheless, higher sequencing depth means higher cost. What is more, increasing sequencing coverage is not always effective and necessary. The pioneer studies had revealed that the sequencing depth at least four reads were required for calling genotype from homozygous and heterozygous loci to ensure 95% confidence, and depth of 6–89 was recommended to balance the cost and the quality of data. In this regard, the average sequencing depth was 12.15 in the present study, which far above the lower limit. Hence, the SNP genotype used for analysis could provide the most conserved result.

So far, SNP genotyping is still difficult to accomplish. Although the methods, such as capillary electrophoresis, high resolution melting, etc., could be used to differentiate SNP alleles, they depend on a special instrument and test kit. By contrast, the reduction of genome complexity and RAD-seq was the best choice for SNP genotyping [24]. However, it was still expensive now. The practices in rice, wheat and maize have proved DNA microarray is the best choice for SNP genotyping, which was characterized as high throughput, high accuracy, fast and cheap track. In this study, we harvested 38,605 RAD-tags with 832,204 polymorphism SNPs using RAD-seq, which provided useful information and should be helpful in developing SNP chip in the future.

### The Centre of origin of sweetpotato and its wild species *I. trifida*

Currently, two major hypotheses about sweetpotato origins were proposed. One scenario suggests that the domestication of sweetpotato may be originated in Central American based on genetic diversity analysis [25, 26]. And the other regards that two domestication events happened in Central America and northwestern South America, which was supported by a distinct divergence between the Northern and Southern gene pools [6, 26, 27]. In contrast, the origins of *I. trifida* were clear, but its taxonomy has long been controversial. Tamari and Kobayashi found the wild diploid species in Acapulco, Mexico, which was believed to be a typical *I. trifida* now [28, 29]. In the same area as where the diploid species had been collected, triploid Ipomoes seed was also collected by Shiotani et al. in Acapulco, Mexico [30]. Tetraploid Ipomoea closely resembling sweetpotato, were also collected in Mexico, Guatemala, Colombia and Ecuador [31, 32]. And those tetraploids observed from the Andes to Mexico were believed to have been derived from the autopolyploid of diploid *I. trifida* [29]. In 1955, Nishiyama collected a wild hexaploid (6X, 2n = 90) in Fortin, Mexico, and designated the wild plant as *I. trifida* (H.B.K.) G. Don [33], which could be crossed with sweetpotato. Beyond that, several communities of *I. trifida*, including diploid, tetraploid and hexaploid *I. trifida* individuals were observed along the coast of Santa Marta, Maracay and Colombia. These plants are widely distributed in places at elevations of 5 to 20 m, and also could be found in areas of about 1000 m above sea level [29].

All these reports can briefly summarize that *I. trifida* and its relatives located mainly in the belt zone reaching from the Pacific Ocean to the Gulf of Mexico at about 17^o^-20^o^N latitude [34], and the most probable this vast geographical region was one of domestication of sweetpotato. In addition, based on the morphological, cytological and genetical evidence, it is logical to assume that *I. trifida* was one of direct progenitor of the sweetpotato [33, 35–37]. In recent years, numerous genetic, phylogenetic and cytogenetic studies have further confirmed the close relationship between *I. batatas* and *I. trifida* [3, 7, 38]. However, further research is needed to precisely determine when and where and how sweetpotato domestication took place.

### *I. trifida* 6X had a closer relationship with sweetpotato

Two principal hypotheses about the origin of *Ipomoea batatas* were proposed. One proposed that *I. batatas* was autopolyploidization or allopolyploidization from *I. trifida*. The other held that *I. trifida* and *I. triloba* were the wild ancestors of *I. batatas*. Austin (1988) noted that *I. trifida* and *I. triloba* were most likely the ancestors of cultivated sweetpotato [10]. Roullier et al. did not support *I. batatas* was originated from *I. triloba* [6]. They considered that *I. trifida* and *I. batatas* are closely related. Most of researchers accepted that *I. trifida* was one of the ancestors of *I. batatas*. However, there is no agreement about the formation of the *I. batatas* polyploid genome whether autopolyploid originated from *I. trifida* or allopolyploidization from *I. trifida* and distant relative species. Kobayashi et al. pointed out that an autopolyploid of sweetpotato was from the ancestor. It shares with *I. trifida* [29].

Four different polyploidy types of *I. trifida* were found, including 2X (2n = 2X = 30), 3X (2n = 3X = 45), 4X (2n = 4X = 60) and 6X (2n = 6X = 90). It is important to reveal the relationship between sweetpotato and different polyploidy types of *I. trifida*. The evolution analysis using SNP genotypes, which distributed the whole genome of sweetpotato and *I. trifida*, should be more reliable and accurate than traditional molecular markers. Three different polyploidy types of *I. trifida* and cultivated *I. batatas* formed well separated clusters. Within the cluster of *I. trifida*, 3 different polyploidy types were grouped into three distinct lineages. Both nested within Beinong5521 and *I. trifida* 6X accessions were intermediate between the cultivated sweetpotato and the *I. trifida* 2X clusters. Among the polyploid *I. trifida* accessions distribution in our evolutionary tree, although they had different polyploidy and different regions, they had a close genetic relationship. It could propose at least one polyploidization or hybridization events of *I. trifida* 2X generated *I. trifida* 4X. On this basis, another two or more polyploidization or hybridization events generated *I. trifida* 6X.

Interestingly, the location of *I. trifida* 6X was far from *I. trifida* 2X, and between *I. trifida* 4X and sweetpotato on the evolutionary tree. It has a closer genetic relationship with sweetpotato than with *I. trifida* 4X and *I. trifida* 2X. This result supported Nishiyama’s hypothesis [34], and should be valuable to further clarify the origins of sweetpotato. Kobayashi et al. reported that the wild polyploids of *I. batatas* were collected from Mexico to northern Peru [29]. Furthermore, our hybridization studies lasting more than 3 years showed that the crossing of sweetpotato with *I. trifida* 6X was more easy to succeed than with *I. trifida* 2X or *I. trifida* 4X, which supported that sweetpotato had more closer genetic relationship with *I. trifida* 6X than with diploid *I. trifida*. However, further study using whole-genome sequencing and other advanced biotechnology to reveal the origins of sweetpotato is needed.

### Functional annotation of sequence with SNPs

A total of 11,519 RAD-tags with SNPs loci were matched unique genes in public databases, which only counted 29.90% of total. Most of them were assigned to different function types of gene ontology categories and COG classifications, including ‘Signal transduction mechanisms’ ‘Posttranslational modification, protein turnover, chaperones’ and ‘Defense mechanisms’, etc.. From blasting in KEGG databases, about 1727 related genes were hit, which connected to the 349 well represented pathways, such as ‘Starch and sucrose metabolism’, ‘Carotenoid biosynthesis’, ‘Brassinosteroid biosynthesis’ and ‘Plant hormone signal transduction’, etc.. Therefore, these results preliminarily showed these RAD-tags related to different functional genes, and they should be useful in functional gene studies. Compared to transcriptome sequencing, the RAD-seq could generate more SNP genotype and acquired more abundant genome information. In addition, highly similar species of *I. batatas*, *I. nil* and *I. trifida* were found in the NCBI dataset, suggesting the presence of an orthologue in the sweetpotato genome.

## Conclusions

In conclusion, our study results suggested RAD-seq should be more efficient and reliable than traditional molecular methods in evolution and genetic study of sweetpotato and *I. trifida*. Thousands of SNP were detected from RAD-seq and annotated with public datasets. Based on SNP genotypes, the evolution relationship between sweetpotato and different polyploidy wild species *I. trifida*, a putative wild ancestor of sweetpotato, was revealed for the first time that cultivated sweetpotato has the closest genetic relationship with *I. trifida* 6X, closely followed by *I. trifida* 4X. In contrast, *I. trifida* 2X has a further genetic relationship. The result provided a valuable clue for researchers to use *I. trifida* 6X as the model plant of sweetpotato research, which should be more practical than using *I. trifida* 2X in the future. Meanwhile, SSR primers, designed from *I. trifida* 6X, should be helpful to solve the problem of lacking SSR markers in *I. trifida* study.

## Methods

### Plant materials

Thirteen sweetpotato accessions, including 4 foreign varieties, 4 Chinese varieties and 5 Chinese landraces, 12 different polyploidy *I. trifida* accessions containing diploid types, tetraploid types and hexaploid type, and 2 genotypes from protoplast fusion were used in the present study (Table 4).

**Table 4:** The sample names, taxonomic, polyploidy and the origin of 27 accessions used in present study

Sample	Taxonomic	Polyploidy	Origin
*I. trifida* 2X B1	*Ipomoea trifida*	2 N = 2× = 30	Mexico
*I.* Trifida 2X Y23	*Ipomoea trifida*	2 *N* = 2× = 30	Unknown
*I.* Trifida 2X Y22	*Ipomoea trifida*	2 N = 2× = 30	Unknown
*I.* Trifida 2X 6.1–2	*Ipomoea trifida*	2 N = 2× = 30	Unknown
*I.* Trifida 2X E3	*Ipomoea trifida*	2 N = 2x = 30	Costa Rico
*I.* Trifida 2X A4	*Ipomoea trifida*	2 N = 2x = 30	Venezuela
*I.* Trifida 2X D1	*Ipomoea trifida*	2 N = 2x = 30	Mexico
*I.* Trifida 2X 13.1–4	*Ipomoea trifida*	2 N = 2× = 30	Unknown
*I.* Trifida 4X 20.3–19	*Ipomoea trifida*	2 N = 4× = 60	Unknown
*I.* Trifida 4X 6.1–16	*Ipomoea trifida*	2 *N* = 4× = 60	Unknown
*I.* Trifida 4X 20.3–16	*Ipomoea trifida*	2 N = 4× = 60	Unknown
*I.* Trifida 6X	*Ipomoea trifida*	2 *N* = 6× = 90	Unknown
Beinong 5521	Synthetic	2 *N* = 5× = 75	Synthetic
Beinong 6–13	Synthetic	2 *N* = 6× = 90	Synthetic
Dalanguo	*Ipomoea batatas*	2 N = 6× = 90	Chinese local variety
Liushiri	*Ipomoea batatas*	2 N = 6× = 90	Chinese local variety
Jinhuanggua	*Ipomoea batatas*	2 N = 6× = 90	Chinese local variety
Shengwuyan	*Ipomoea batatas*	2 N = 6× = 90	Chinese local variety
Yongchunwuchi	*Ipomoea batatas*	2 N = 6× = 90	Chinese local variety
Qingpizhong	*Ipomoea batatas*	2 N = 6× = 90	Chinese local variety
Baiguqilong	*Ipomoea batatas*	2 N = 6x = 90	Chinese local variety
Xiangyushu	*Ipomoea batatas*	2 N = 6x = 90	Chinese local variety
Xushu23	*Ipomoea batatas*	2 N = 6x = 90	Chinese modern variety
Chuanshu217	*Ipomoea batatas*	2 N = 6x = 90	Chinese modern variety
Xushu27	*Ipomoea batatas*	2 N = 6x = 90	Chinese modern variety
Guangshu85–108	*Ipomoea batatas*	2 N = 6× = 90	Chinese modern variety
Gaoshu17	*Ipomoea batatas*	2 N = 6× = 90	Chinese modern variety

### DNA extraction

CTAB (cetyltrimethyl ammonium bromide) method was used to isolate genomic DNA from all samples [39]. The concentration of all DNA samples was quantified using NonoDrop ND-2000 (Thermo Scientific, Wilmington, DE, USA) and confirmed by 1% agrose gel electrophoresis. Finally, the original DNA was dissolved in 1 × TE buffer (10 mM Tris-HCl and 1 mM EDTA, pH 8.0), and diluted to 50 ng/ul with ddH_2_O for RAD analyses.

### RAD libraries construction and sequencing

A total of 27 RAD libraries were prepared following the method described by Baird et al. [19]. First, enzymes *EcoRI* and *NlaIII* were chosen from comparison of the number of repeated tags and the distribution of enzymatic tags of different enzymes. Second, RAD libraries were constructed using pre-selected enzymes. Then, enzymolysis genomic DNA of all samples ligated with the P1 adaptor, which containing Illumina sequence primers, PCR forward primers and barcodes, were pooled together. 300-400 bp fragments were isolated using agarose gel electrophoresis and purified. Retrieved DNA fragments were ligated with another adapter (P2), which including PCR reverse primers and divergent ends. Finally, DNA fragments with two adapters (P1 and P2) were selectively amplified through PCR reactions. PCR products were purified and pooled, then separated on a 2% agarose gel. Fragments with 375-400 bp (with indexes and adaptors) in size were divorced and purified. Pair-end (PE) sequencing was performed using an Illumina HiSeq4000 platform (http://www.illumina.com/). Quality filtering and loci assembly were conducted utilizing Stacks v1.40 [40]. The sequences of each sample were sorted depending on the barcodes.

To ensure the nucleotides quality value above Q30 (< 0.1% sequencing error) and more than 99% above Q20 (< 1% sequencing error), raw reads with ≥10% unidentified nucleotides (N), > 50% bases having phred quality < 5, with > 10 nt aligned to the adapter, containing enzyme sequence, were discarded. Raw sequence reads with RAD-tag were divided following the order of sequence depth. Heterozygous loci were tested through comparison in each sample. The comparison among RAD-tag of discrete samples produced SNPs. Finally, combine the frequency data and comparison results, low confidence SNPs from repeat regions were filtered.

### SNP detection and annotation

Single nucleotide polymorphisms (SNPs) were marked with maximum likelihood models, implemented in Stacks v1.40 [40]. For SNPs calling, all trimmed reads were collapsed into clusters based on sequence similarity using Stacks v1.40 [40] under default parameters. Vcftools v 0.1.1.12b [41] was used for quality control. Finally, SNP loci detected more than 22 individuals and with allele frequency greater than 0.1 were used for further analysis.

To avoid mistakes, RAD-tags depth above 500 were excluded. Blast was done with sequences of RAD-tags of all samples. SNPs were identified in alignment results, and regarded as true polymorphisms when each allele was observed at least four times. Genotypes of all samples were determined by the resultant sequence reads containing SNPs loci.

To assess the potential function of the SNP loci, a similarity search were conducted using local gene finding software Blast2GO v2.4.2 [42] with E value cutoff of 10^− 5^ against Non-redundant (Nr) database, Gene Ontology (GO) database, Cluster of Orthologous Groups of proteins (COG) (http://www.ncb*I.*nlm.nih.gov/COG) database, Kyoto Encyclopedia of Genes and Genomes (KEGG) database (http://www.genome.jp/kegg) and the UniProt Knowledgebase (UnProtKB) protein database. RAD sequences contained SNP loci were aligned with these five databases to predict and classify possible functions.

### SSR markers development

MIcroSAtellite (MISA, http:// pgrc.ipk-gatersleben.de/misa/) script was used to detect microsatellites in RAD-tags with default parameters. The SSR (Simple sequence repeat) loci with repeat units of 2–6 nucleotides and at least five reiterations in each repeat unit were retained for designing primers. Then, the tags containing microsatellites were used for primer design using Primer premier 5.0 (PREMIER Biosoft International, Palo Alto, CA) following the default setting excluding three criteria [43, 44], (1) the length of primer were ranged from 18 to 25 bases, (2) GC content of primers within 40–60% and the annealing temperature between 50 °C and 60 °C, (3) PCR products size of designed primers was 100 bp–400 bp.

In order to make sure whether these primers worked, 68 primers were randomly synthesized and amplified in 8 *I. trifida* accessions. The condition of PCR amplification and electrophoresis as described Ban et al. [45]. The Fragment Analyzer INFINITY™ (Advanced Analytical Technologies (AATI), Ames, Iowa, USA) electrophoresis system and DNF-900 dsDNA Reagent Kit were used to separate PCR products, and the results were analysed by PROSize 2.0 Software Version 1.3 (AATI).

### Evolutionary analysis of sweetpotato and *I. trifida*

Neighbor-joining phylogenetic tree was undertaken using Phylip (v 3.695) [46] based on SNP dataset after quality control. The Neighbor-joining phylogenetic tree was generated with R-package of APE and iTOL v3 (http://itol.embl.de) [47, 48]. On the premise of the K value of the subgroup number from 0 to 10, population structure was inferred using fastSTRUCTURE (v1.2) with the default parameters [49]. K with marginal maximum likelihood from each K analysis was selected as the optimal subgroup number. Genetic similarity matrix was calculated between each sample using PLINK (v1.90) [50, 51] based on filtered dataset. Then, the tree of unweighted pair group method of arithmetic mean (UPGMA) clustering was calculated with Phylip (v 3.695) [46] from genetic similarity matrix, and draw with R-package of APE [48] and iTOL v3 (http://itol.embl.de) [47].

## Additional files

Additional file 1: **Figure S1.** The distribution of reads number in different average fragments depth. Figure S2. The distribution of Top-hits species based on Gene Ontology (GO) result. Figure S3. The sequence distribution with different Evidence Codes. Figure S4 COG classification assigned sequences to top orthologous groups. The x-axis represents the abbreviation of COG Categories, and the full name of COG Categories was on the right. The y-axis denotes the sequence number. Figure S5. The sequence distribution in different pathway detecting from the KEGG database. (PDF 1490 kb)

Additional file 2: **Table S1**. The distribution of SSR loci with different SSR unit and different unit number. (DOCX 27 kb)

## Abbreviations

BA: Biological aspect of ancestor
BP: Biological process
CC: Cellular component
COG: Clusters of orthologous groups of proteins
CTAB: Cetyltrimethyl ammonium bromide
DA: Direct assay
DNA: Deoxyribonucleic acid
EA: Electronic annotation
EP: Expression pattern
GBS: Genotyping-by-sequencing
GO: Gene ontology
KEGG: Kyoto encyclopedia of genes and genomes pathway
MF: Molecular function
MISA: Microsatellite
MP: Mutant phenotype
NCBI: National center for biotechnology information
NGS: Next-generation sequencing
Nr: Non-redundant
PCR: Polymerase chain reaction
PE: Pair-end
RAD-seq: Restriction site-associated DNA sequencing
RRLS: Reduced-representation libraries sequencing
SM: Sequence model
SNP: Single nucleotide polymorphisms
SS: Sequence or structural similarity
SSR: Simple sequence repeat
UnProtKB: UniProt Knowledgebase
UPGMA: Unweighted pair group method with arithmetic mean clustering
