# Supplementary material for: Analysis of evolution and genetic diversity of sweetpotato and its related different polyploidy wild species I. trifida using RAD-seq
Source: BMC Plant Biol. 2018 Sep 5;18:181. doi: 10.1186/s12870-018-1399-x (PMC6126004; doi:10.1186/s12870-018-1399-x)
Supplement: Supplementary file 1 — Figure S1. The distribution of reads number in different average fragments depth. Figure S2. The distribution of Top-hits species based on Gene Ontology (GO) result. Figure S3. The sequence distribution with different Evidence Codes. Figure S4 COG classification assigned sequences to top orthologous groups. The x-axis represents the abbreviation of COG Categories, and the full name of COG Categories was on the right. The y-axis denotes the sequence number. Figure S5. The sequence distribution in different pathway detecting from the KEGG database. (PDF 1490 kb) [file 12870_2018_1399_MOESM1_ESM.pdf]

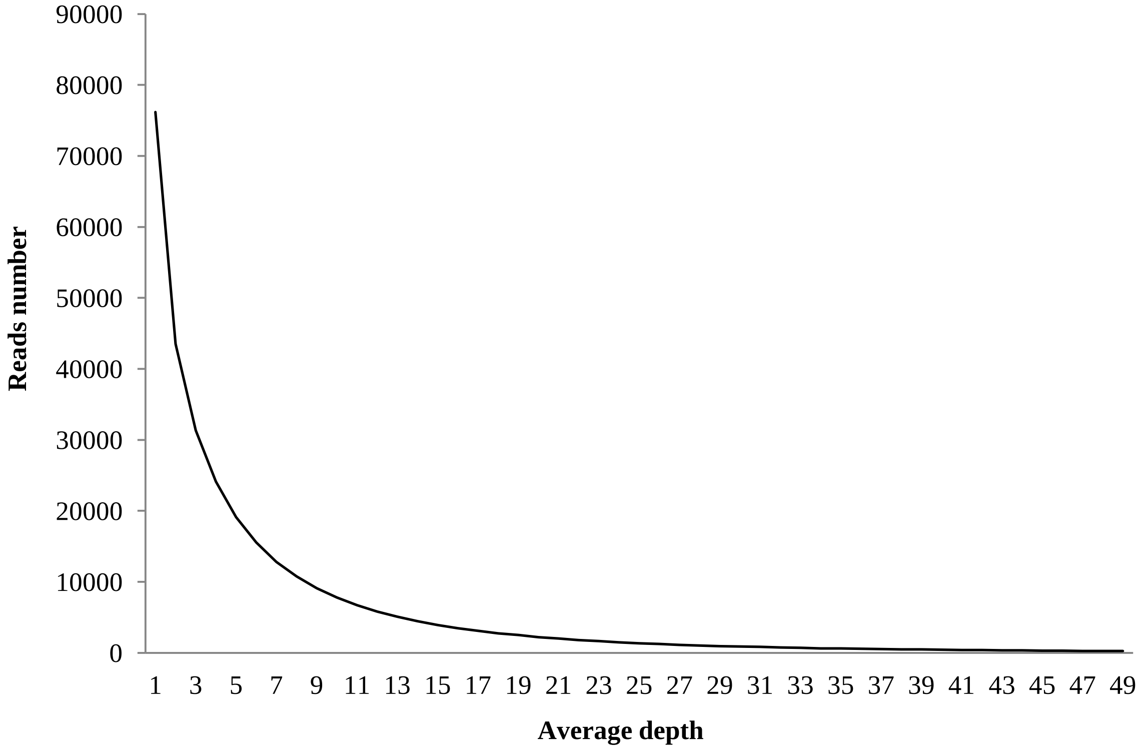

Figure S1 The distribution of reads number in different average fragments depth

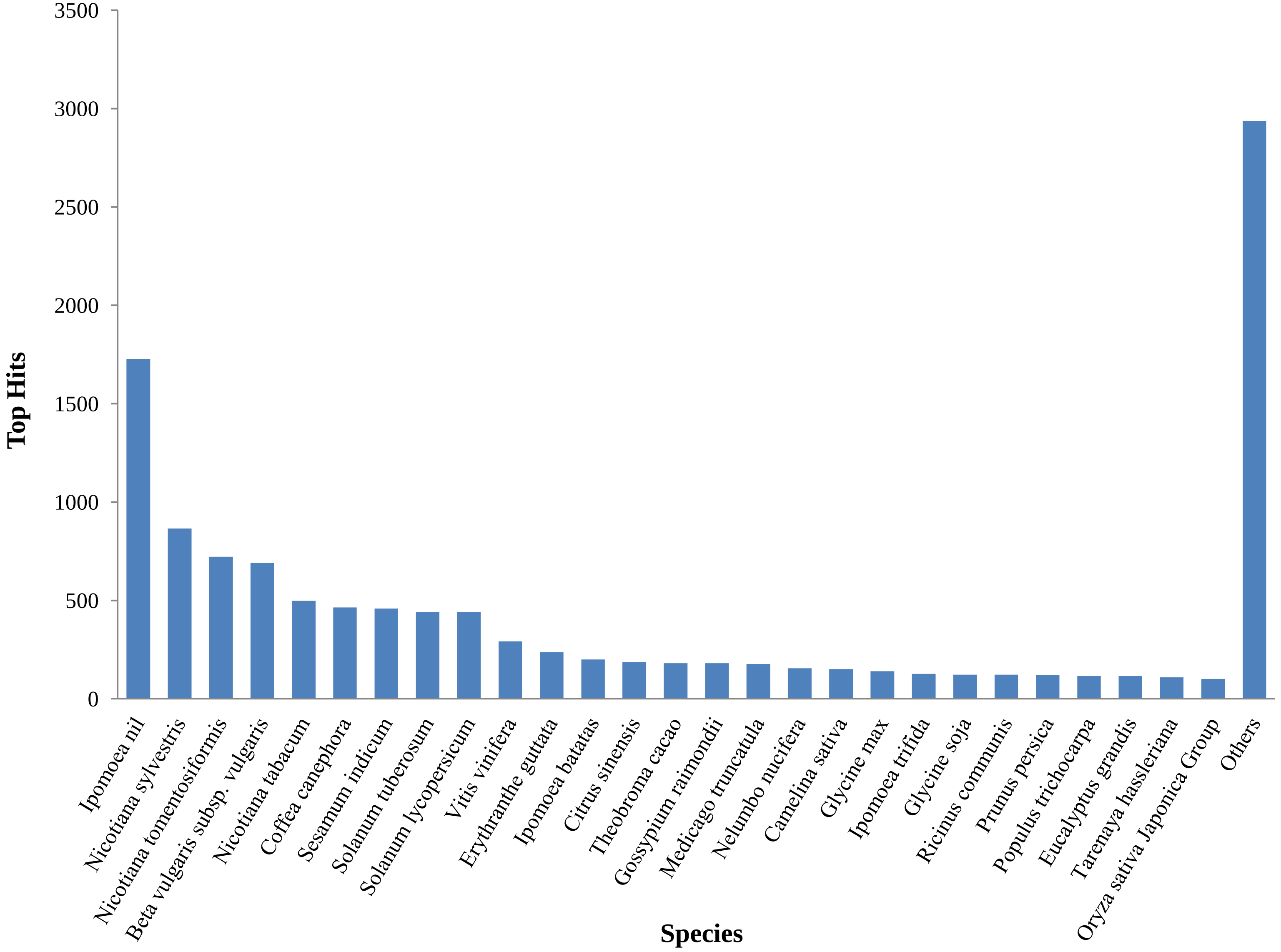

Figure S2 The distribution of Top-hits species based on Gene Ontology (GO) result

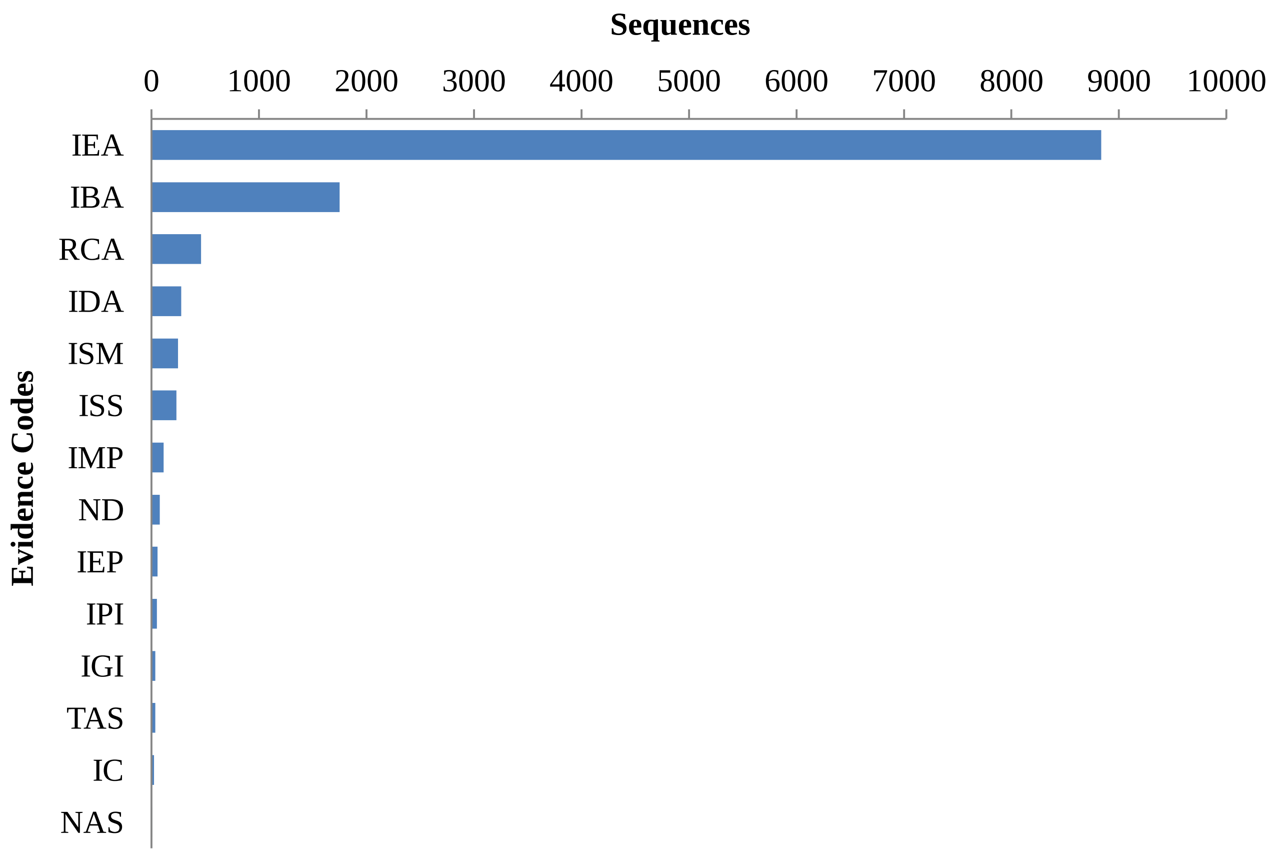

Figure S3 The sequence distribution with different Evidence Codes

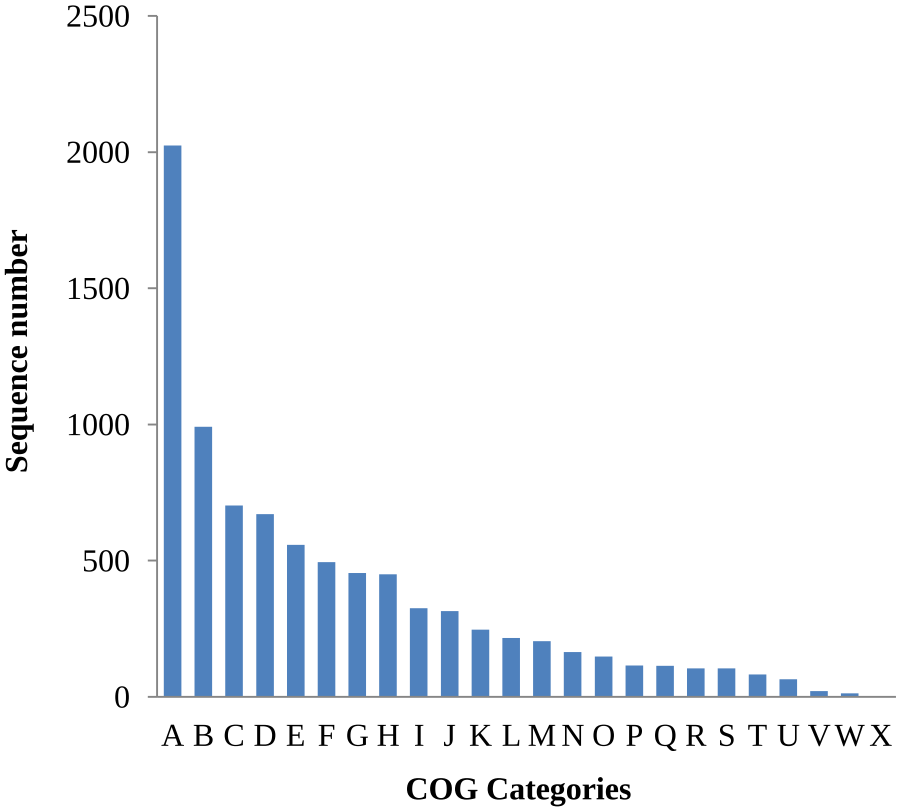

A Function unknown
B Signal transduction mechanisms
C Posttranslational modification, protein turnover, chaperones
D Energy production and conversion
E Replication, recombination and repair
F Carbohydrate transport and metabolism
G Intracellular trafficking, secretion, and vesicular transport
H Secondary metabolites biosynthesis, transport and catabolism
I Transcription
J Amino acid transport and metabolism
K Translation, ribosomal structure and biogenesis
L Lipid transport and metabolism
M Inorganic ion transport and metabolism
N Defense mechanisms
O Cell cycle control, cell division, chromosome partitioning
P Cytoskeleton
Q Cell wall/membrane/envelope biogenesis
R RNA processing and modification
S Nucleotide transport and metabolism
T Coenzyme transport and metabolism
U Chromatin structure and dynamics
V Nuclear structure
W Extracellular structures
X Cell motility

Figure S4 COG classification assigned sequences to top orthologous groups. The x-axis represents the abbreviation of COG Categories, and the full name of COG Categories were on the right. The y-axis denotes the sequence number

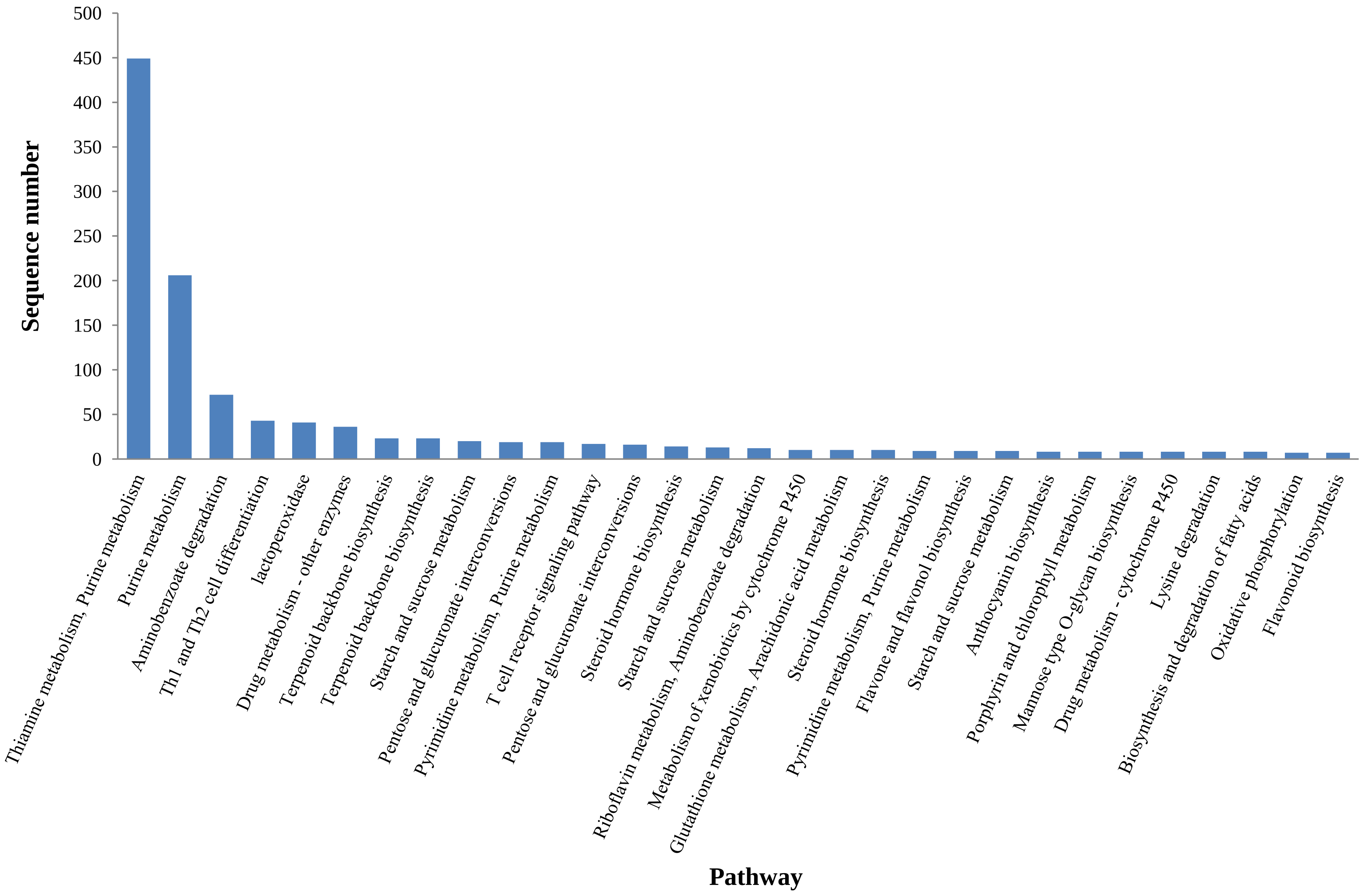

Figure S5 The sequence distribution in different pathway detecting from KEGG database
