# Supplementary material for: Analysis of evolution and genetic diversity of sweetpotato and its related different polyploidy wild species I. trifida using RAD-seq
Source: BMC Plant Biol. 2018 Sep 5;18:181. doi: 10.1186/s12870-018-1399-x (PMC6126004; doi:10.1186/s12870-018-1399-x)
Supplement: Supplementary file 2 — Table S1. The distribution of SSR loci with different SSR unit and different unit number. (DOCX 27 kb) [file 12870_2018_1399_MOESM2_ESM.docx]

**Table S1** The distribution of SSR loci with different SSR unit and different unit number

| Unit number | Unit with 2 bases | Unit with 3 bases | Unit with 4 bases | Unit with 5 bases | Unit with 6 bases |
| --- | --- | --- | --- | --- | --- |
| 5 | - | 1,067 | 171 | 43 | 45 |
| 6 | 1,128 | 411 | 38 | 15 | 15 |
| 7 | 621 | 158 | 20 | 4 | 2 |
| 8 | 396 | 73 | 10 | 0 | 2 |
| 9 | 232 | 57 | 8 | 0 | 0 |
| 10 | 146 | 39 | 6 | 0 | 0 |
| 11 | 93 | 17 | 0 | 0 | 0 |
| 12 | 59 | 16 | 0 | 0 | 0 |
| 13 | 33 | 6 | 0 | 0 | 0 |
| 14 | 29 | 8 | 0 | 0 | 0 |
| 15 | 13 | 5 | 0 | 0 | 0 |
| >16 | 29 | 25 | 2 | 0 | 0 |
